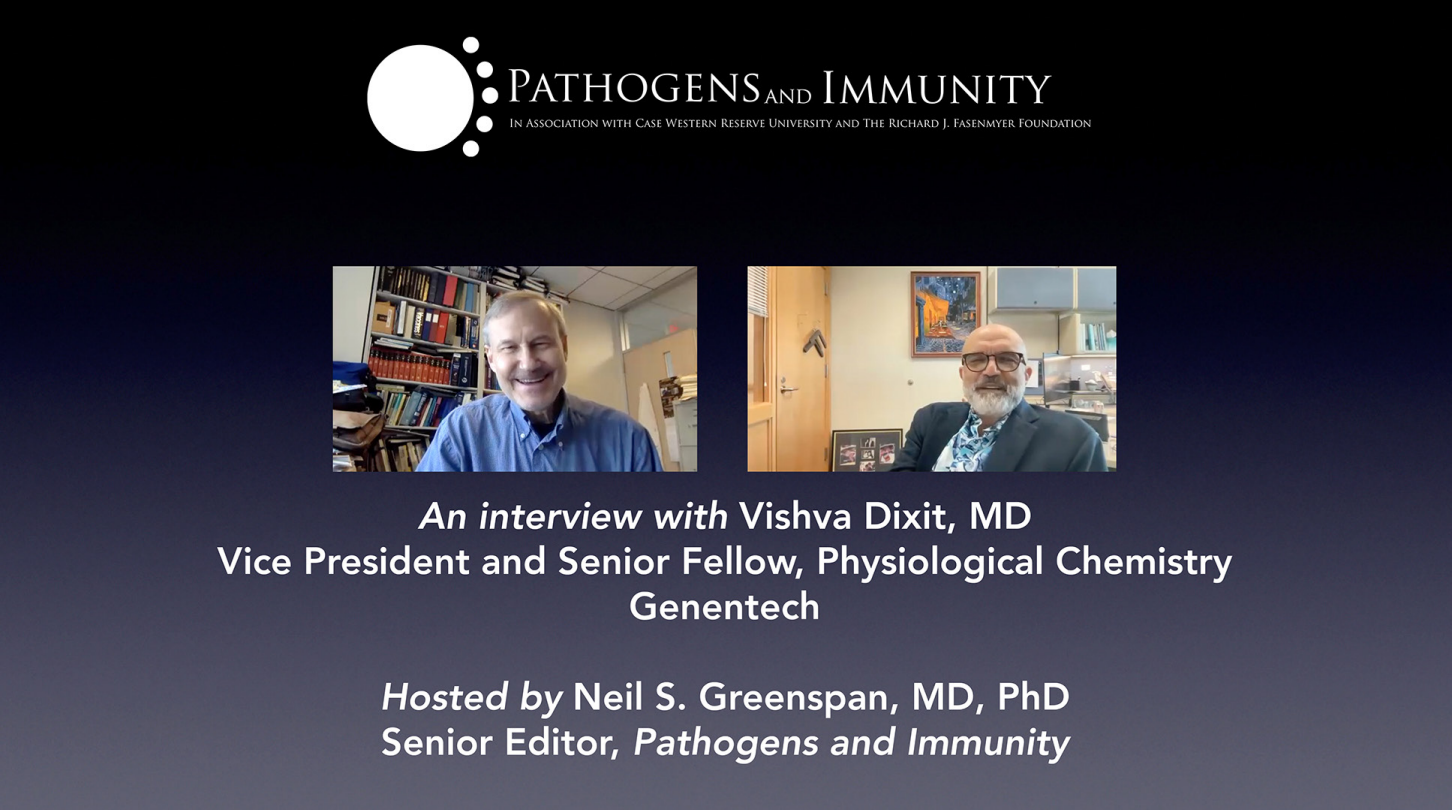# Vishva Dixit Shares Insights on His Early Influences, Research Career, Key Findings, and What it Takes to Be Successful in Science

**DOI:** 10.20411/pai.v8i2.643

**Published:** 2023-11-20

**Authors:** Neil S. Greenspan

**Affiliations:** 1 Case Western Reserve University, Cleveland, Ohio

*This interview has been edited for clarity.

In this interview, Vishva Dixit, MD, talks with Neil S. Greenspan, MD, PhD, senior editor of *Pathogens and Immunity* about what his career in science has revealed about biology in humans, but also what it has revealed about how to be successful in science. Dr. Dixit is a world-famous immunologist and biochemist who has made major contributions to the study of innate immunity in general and particularly to understanding inflammasome activation. He is vice president and senior fellow in physiological chemistry at Genentech.

## NEIL S. GREENSPAN, MD, PHD

Can you talk about where you grew up, where you went to school, and the various stages in your career in biomedicine and biomedical research?

## VISHVA DIXIT, MD

I was born and brought up in Kenya, in East Africa. And this was, during colonial times. At that time, Kenya was a British colony, and it was another world. There was a very distinct color line and segregation of races as sort of in an apartheid system. Independence came in 1963 with the integration of schools, and that was also, at a certain level, socially traumatic. But I proceeded to go through the school system there, and then attended medical school at the University of Nairobi, which I left in 1981 to begin a residency in laboratory medicine, at Washington University in St. Louis.

## NG

From my understanding, in your very early years, you were in the more rural and isolated parts of Kenya and, later, more in Nairobi, the main city. Did your experiences in Kenya and then in the United States influence how you think your intellectual development unfolded?

## VD

I think I was, in many ways, blessed to have been exposed to such a broad spectrum of society. In Kenya, my parents were both physicians that practiced in a small town in the highlands. That was a very different existence for the Kalenjin people. You have to realize that the people that they served were very poor. But, when my father passed away in Nairobi after a long illness from pancreatic cancer, I was really surprised, shocked, and in disbelief that the villagers had collected money and paid off his hospital bill to thank him for the services he had rendered [to] the community over the decades. So that underscored to me that, even though they're [from] very different cultural settings, that at our very core, our values, aspirations are often quite similar. Unfortunately, they're exaggerated, and they're given an “us versus them” spin by politicians and religious preachers. And that's done as an instrument for exerting power. The other fact that I took away from my experiences is that while education is key for the betterment of societies, it really has to be coupled with the respect for differences and, indeed, in many ways, diversity is to be celebrated. But, at the end of the day, it suggested that we are more similar than we are different.

## NG

It's both a remarkable testament to your father and of how he devoted himself to the patients that he treated. But also, your comments, although not exactly what *Pathogens and Immunity* normally focuses on, are well received and, I think, quite insightful. Can you recount what early influences steered you to biomedical science as opposed to clinical practice?

## VD

Growing up in the small town [of] Kericho, in the highlands of Kenya, I was a curious kid. But my vision was seriously compromised and that precluded any participation in sports. But I could read, and so I buried myself in books. And I was greatly enamored by the explorers of old, those who had discovered new lands and treasures. Somewhere along the way, I realized that much of that had already been discovered. But there was an equally compelling unknown to be explored in the sciences. I mean, you could be an explorer in the modern world as a scientist and have the joy of discovery. I think that was the seed that said to me that if you want to be an explorer in the modern-day world, be a scientist.

## NG

That's quite beautiful. Looking back on your highly successful career, can you identify one or more decisions that had a major impact on what followed and that you believe facilitated your career progress?

## VD

Gaining admission to be a resident in the storied Department of Pathology at Washington University in Saint Louis. This was, of course, greatly facilitated by my brother, who was a fellow in rheumatology. He convinced the department to give me a chance and take me as a resident in lab medicine. And, for me, it was just a wondrous and eye-opening experience. Not only was their emphasis on research but, most surprisingly for me, knowledge was continually questioned in journal clubs and research meetings. And presenting at these forums that we used to refer to as the Shark Tank was a daunting experience, and, in hindsight, it was probably the experience that was most valuable to me. Because not only did you have to know your material, but you had to address a stream of penetrating questions from really astute minds in the audience. It really made you think on your feet. For me, it was a watershed moment. For the first time I really understood what it takes to be a researcher – that one has to be critical. Critical of one's own work and critical of the work that you read about, critical of the work of others. So that was probably the most enlightening experience I had in my career; was being at Wash U in St. Louis. It was just an amazing time.

Sometimes I think back to those meetings, and I'm not sure that sort of tenor of questioning and challenging would be allowed today, at least it would be frowned upon, but I think it's a pity, because I think it's so important to be challenged and to learn from that. So that, for me, is that sort of crucible of knowledge that allows you to be a successful researcher.

## NG

What do you regard as your most significant discoveries?

## VD

I would say the first one had to do with receptor signaling. We were very interested in how death receptors like FADD (Fas-associated death domain) and TNF (tumor necrosis factor) receptors signaled. And at the time we embarked on our studies, receptors were thought to signal by either altering phosphorylation/dephosphorylation events or by functioning as ion channels. But we uncovered a third way that receptors can signal, and this was through the recruitment and activation of a protease. So, in other words, the second messenger was a protease, which was quite unexpected. And this is a common way of signaling with death receptors. They activate these proteases, termed caspases, cysteine proteases that cleave after an aspartic acid residue. But the other thing that fell out of the study of these signaling complexes was the realization that they were held together by a protein-protein interaction motif that was dubbed the death domain. And so we started looking at databases for other examples of death domains. And we discovered a lot of interesting molecules that had death domains, but I'll just highlight one of them, which was MyD88 (myeloid differentiation primary response 88 and other similar names), and we discovered it had a death domain. And MyD88 was the conduit for signal transduction from the IL-1 receptor, and later it was shown to be the conduit from TLR (Toll-like receptors). So that frenzy of looking for molecules with the death domain turned out to be very fruitful. And in that search, we also uncovered the rest of the mammalian death proteases, these caspases, and so that was a very fruitful period. It happened mostly during my time at Michigan. But once I came to Genentech, this would be in 1997 or so, we became more interested in the inflammasome. And so, this would be a pathway that regulates inflammatory caspases such as caspase-1 and leads to the proteolytic maturation of IL-1 beta and IL-18. And during this work, we made I think, quite unexpected discoveries. So, in 2011, the Nobel Prize had been given for the LPS (lipopolysaccharide) receptor, which was Toll-like receptor 4. One assumed that was the way by which LPS signaled. But, in 2013, we discovered that LPS could signal through a TLR4-independent manner, and in doing so, activated what we called the noncanonical inflammasome. So, this was unexpected, because we went on to show that at high-dose LPS, TLR4 KO mice could be sensitive to killing by LPS. So TLR4 was dispensable. But what was required was this noncanonical pathway. This of course, has ramifications for cytokine storm and sepsis. And in studying this noncanonical pathway of LPS responsiveness and the approach was mostly forward genetics in mice, we inadvertently discovered how leaderless cytokines are released. So leaderless cytokines are cytokines like IL-1 alpha, IL-1 beta, IL-18, [and] IL-33. These are very important pro-inflammatory cytokines. And they are actually released through a membrane channel that's formed by a protein, gasdermin D. So, this was a problem that cell biologists had pondered for a long time. And, in the end, the solution was quite simple. When there are inflammatory insults and the inflammasome is activated, you get the creation of a pore, and this allows for the leakage of leaderless cytokines to the exterior. And then this led to our most recent discovery, that of NINJ1, which is a discovery that violates what we were taught by our high school biology teachers, because what we were taught is that following cell death, cell lysis is a passive osmotic process. But we actually found that there's a membrane protein that greatly accelerates cellular lysis. So, this was quite unexpected and, again has important ramifications for innate immunity and cell biology in general. That's a quick overview of decades of work. But as you can see, the central theme is [cell] death and inflammation and their relationships.

## NG

That's fascinating. It's interesting, too, and it's quite a typical example, where domains are called death domains and then it turns out they're in proteins that don't really deal in death, per se. It reminds me of an example I once came across, and I've written about a little bit. As you might expect, dung beetles normally survive by gaining their nutrition through the consumption of dung. But a group, I can't remember where they were doing this, it might have been South America, discovered a beetle that was extremely similar in all its morphological features to every other dung beetle, but it didn't eat dung. And so, with examples like that, I formulated what I call the principle of radical evolutionary indifference, which says that evolution doesn't care about our categories and preconceptions.

## VD

Oh, absolutely. That's a great example of that.

## NG

Your retelling of just a few highlights from your career illustrates how one question leads to another and another. And it's so often the case that assumptions that have been made get overturned when you look more closely. Beautiful examples. Considering what you've just described, do you have any particular approach to identifying problems or questions you think are worth pursuing?

## VD

You know, again, I was greatly influenced by what I learned at Wash U. One was not to enter a field that is crowded, because it's then very difficult to make a dent and maybe think more unconventionally, against the grain. And I think you have to find out, what's your comfort? What do you enjoy? What's your temperament? And I really like what I would call 0-to-1 discoveries, finding something new rather than taking something from 1 to 100, which is more consolidation and refinement. But the way I decide on questions is to think about them, but really discuss them very extensively with my colleagues and discuss them with a view that they play devil's advocate. So, we really want to pressure test the idea, because that is so important. One can be deluded into one's own ideas and thinking that they're the greatest thing since sliced bread, but I think just having those discussions and having a cadre of colleagues who were critical, I have found is important. Once it passes that pressure test, then we will do some exploratory work, and if there is something to it, if it catches traction, then we will focus on it. But I would say that, and we often take a genetic viewpoint – it could be a forward genetic screen, it could be a CRISPR screen. But the foundation is often laid by the genetics and the system.

## NG

Do you think it's fair to say that by taking a genetic approach, you're putting up front the question of whether a given phenomenon or a given gene or protein is important *in vivo*, so that you don't spend time discovering *in vitro* phenomena that may never translate into what goes on in the living?

## VD

I think so – and I may be wrong here – the tools of biochemistry are so enormously powerful, that one can show a lot of things in a cell free *in vitro* system that may have no bearing to the living organism. And so, we prefer to do it in reverse, we want to first make sure that it is of critical importance, and then work out the biochemistry.

## NG

Is that primarily the result of your personal approach? Or your personal preferences? Or is it something that is more appropriate for your current environment, which is a for profit company, than let's say if you were still at a university?

## VD

No, I think I've always felt that. I mean, clearly we change with experience, but my experiences have been that the work I've been proudest of, and the work that has stood the test of time, largely has been work that started off with genetics. And then we built on that – the discovery of gasdermin D, the discovery of NINJ1, the discovery of the noncanonical inflammasome pathway. Those were all very intimately linked to a genetic interrogation of the pathways.

## NG

Let's move on to another question that has to do with broader perspectives on immunity and inflammation. Do you accept the hypothesis that the immune system responds to non-self or the competing hypothesis that it responds to so called danger, which has been very much sort of diffusing into the immunology community now for decades? Everybody talks about danger and danger signals. I have my own views on this question, but I'm curious how you think about.

## VD

I've been compelled with the earlier work of Charlie Janeway, who and from that, derived the existence of PAMPs (pathogen-associated molecular patterns) and DAMPs (damage-associated molecular patterns). And one way to think of them is they're means to sound the inflammatory alarm, and that's why they're required as adjuvants in a vaccine. PAMPs and DAMPs certainly alert the immune system, but DAMPs arise from within. And I think it gets a bit semantic, so uric acid crystals are a DAMP. Are they really non-self? Well, I mean, that's debatable. You could say, well, uric acid crystals are self, because you can't have uric acid crystals then deposition and not have gout. Or you could say over a certain threshold, they represent non-self. But I think the basic premise is that there is a system to recognize foreign and internal molecules, the so called PAMPs and DAMPs, and this revs up the innate immune system and is the basis for adjuvant use in vaccines. I mean, that's the way I look at it.

## NG

I just will mention that there are vaccines that don't have adjuvants, although Charlie Janeway is famous for what he called his dirty little secret, that adjuvants are necessary. They certainly can increase responses a lot and make vaccines more likely to be useful. But there have been successful vaccines that don't use adjuvants. And, I just think, at times, the way the word danger gets used, it ends up being almost a tautology, so that if there's a response, it's called the danger signal. And I think there are areas in immunology, where it's very clear that those kinds of signals matter, but they're not the only ones that matter. Another way to put it is, I guess, that I don't think the evolutionary imperatives require that there be only a single dichotomous way for the immune system to make a decision.

## VD

I'd agree.

## NG

Have you ever investigated complement? You mentioned the alternative pathway.

## VD

You know, we haven't really studied it, except to note that they're both protein cascades with built in amplification systems and both are subject to complex regulation, because if they go awry, they can do serious damage. And, ultimately, when they run their full course, they compromise membrane integrity. So, there are those sorts of similarities between these two cascades. But we ourselves have not studied complement activation.

## NG

I didn't think you had. But I just thought it's interesting, because I was recently at a local inflammasome meeting, and they brought in some first-rate people, some of whom were talking as if the study of innate immunity began with Charlie Janeway. I pointed out that the study of complement goes back a century, and not in any way to diminish the importance of the more recent work on inflammasomes and related systems that you've so beautifully delineated in our own discussion today, but to make the point that it is really astonishing how many analogies there are in terms of protein-protein interaction leading to protease activation and signals, and devolving from there. And, it doesn't replace what work has been going on studying intracellular systems, but it's fascinating that there's an extracellular system that is enormously similar, and as you pointed out, ends up with at least one of the same outcomes, which is the destruction of membrane integrity and the amplification of various inflammatory pathways.

## VD

Absolutely. Evolution never ceases to amaze. Bacteria-encoded gasdermin – this was just discovered last year by a group in Boston – and that gasdermin is encoded on an operon that also has a caspase-like protease. So, in the presence of phage, the operon is activated, the caspase-like protease cleaves the bacterial gasdermin, which then forms a pore leading to death of the cell and depriving phage of its replication niche. So, these sorts of cascades that ended up in membrane damage seem to be evolutionarily conserved.

## NG

That's really interesting. I had not come across that.

## VD

It was a paper in *Science* last year.

## NG

It's interesting to consider that CRISPR itself is a bacterial immune response, in a sense.

## VD

Yeah it is. And, you'd say that about restriction enzymes as well. And restriction enzymes and CRISPR are not present in mammalian innate immunity. But certainly, the gasdermins, bridge bacteria to humans, in terms of the conservation of the evolutionary principles of pore formation.

## NG

Is the bacterial gasdermin-like molecule actually similar in primary structure? Or is it just similar functionally?

## VD

It's similar in primary structure. Remarkable.

## NG

That is quite a finding. In your career, have you performed only basic research since you moved to Genentech? Or have you had more product-oriented roles while you've been there?

## VD

So besides running my research lab, I also serve on various committees. Probably the most prominent one is the research review committee that oversees research at Genentech and oversees the early pipeline. We meet twice a week. And it really gives me a bird's eye view of drug development. And at the end of the day, getting a successful drug is nothing short of a miracle. There are just so many slips between the cup and the lip. It's just amazing. It's a committee I've worked on for a long time. It's one that I learn a lot from, and it's a lot of fun. One of the other responsibilities that I have had includes the postdoc program at Genentech. We have about 140 postdocs on campus. And that's just a really enjoyable job, because postdocs at Genentech aren't allowed to work on a product-related project, they have to do basic science, fundamental work. So, it's great to have that environment within a company.

## NG

That's interesting. Is that unusual? I mean, would most companies limit postdocs totally to basic science?

## VD

I think it's unusual at this scale – that we have such a large program devoted to it. It's been in existence for a long time – for 40 years. I would say it's quite unusual to have a program like this. Of course, in more mature industries, like the physics-based industries – IBM, Bell Labs, etc. – there has been a strong tradition of basic research.

## NG

But in terms of other biotech companies?

## VD

I think, fortunately, the good news is that there are companies that are moving in that direction – Novartis, Regeneron come to mind, which have postdoc programs. I think companies are seeing the value of investing in basic research, and I hope that trajectory continues.

## NG

That's a very interesting development, because if the companies recognize the value of the basic science, that's quite impressive given the pressures they face financially. That leads directly into my next question, which is what are your thoughts about the interplay between basic and more practically focused or mission-focused biomedical investigations?

## VD

You know, I think there's an overemphasis in academia and granting agencies on translational research. I really think that these organizations should be fostering basic, fundamental work. You know, we just discussed restriction enzymes and CRISPR and there are other discoveries that were just curiosity driven. And I increasingly get the sense that granting agencies are configured like they're funding engineering projects, they use words like timelines and deliverables. And I don't think that's the best investment of research dollars. And I hope the pendulum begins to swing the other way where there is greater emphasis on curiosity-driven work.

## NG

I expect that people will be taking notice that someone who works at a company believes that academic and granting agencies should focus on basic science and not pretend that they're all about application. Not that there is anything wrong with that, but in the long run, I assume what you're arguing is that investing in the basic science will probably pay off more completely.

## VD

And that is key. And the pharmaceutical biotech industry is built on the foundation of the work done by the NIH, by basic science. So, I think each should do what they do best. And I think the universities shouldn't steer into translational research too deeply at the cost of basic research, because that's where industry is reliant upon the basic research done at universities.

## NG

That's a very interesting perspective. Have any of your specific discoveries yet been transformed into therapeutic or diagnostic applications?

## VD

There are two programs there for which we did a lot of the foundational work. There is this kinase RIPK1 (receptor-interacting protein kinase 1) that is a key kinase in the necroptotic pathway, and then there is a sensor, NLRP3, in the inflammasome pathway that mediates pyroptosis. Inhibitors to both those programs are in the clinic by Genentech, Roche, and other pharma companies. So, we'll wait and see what transpires, but that has been satisfying to see work progress into the clinic from the bench side.

## NG

I'd like to move on to a different topic, which is scientific publishing. What are your thoughts on scientific publishing as it is today, which is probably quite different from when we started?

## VD

I think it's broken. And I think few would argue about that. I think when you look at the history of publishing, it was created to serve a very small community of researchers. It's evident for people who have read “The Eighth Day of Creation,” the birth of molecular biology. If you haven't read it, I would urge your audience to read it. It's a great book. You can appreciate that it was a very small community of researchers that communicated by letters and meetings. But today we have a massive academic industrial complex that spews out thousands of manuscripts a week. So, it's complete exponential growth.

I don't have a ready solution, because the problem is of such great scale – orders of magnitude scale – in such a short time, but I think it's imperative that the community come up with an alternative that satisfies most people, that's a workable model.

## NG

Are you in favor of online publication of manuscripts as preprints, prior to peer review?

## VD

Absolutely, I think it disseminates the research faster. And in many cases that work is funded by the taxpayer. I think it's imperative that it be disseminated to the community.

## NG

I guess I'm referring to entities like bioRxiv and medRxiv. But even if they are published before they have peer review, they attract comments once they are published, so they are not without any commentary.

## VD

It is not without any commentary. Now, I have to say we ourselves have not indulged in that. But I think it's more a reflection of old world thinking on our part. But I see bioRxiv papers all the time, and I appreciate them. I just think that claims could be made in such manuscripts that are incomplete. And, since they are part of the record – correction is always a difficult task.

## NG

Well, as with most human activities, there are pluses and minus. Do you have any preference about anonymous versus open peer review?

## VD

I think that is another one that one struggles with. You can see pluses and minuses again. With anonymity comes the danger of completely unreasonable reviews. And I think you see the danger of anonymity in social media. I think with the open system, comes the danger of earning cookie points. People will review a paper and be known as the reviewer, and then there may be a quid pro quo at a future time. I don't I don't have an answer to that. I've seen it work both ways.

## NG

As you're speaking, I'm thinking that the problem with both is that either one relies on good faith. And there's no way to guarantee good faith 100% of the time in 100% of the people.

## VD

Exactly. It's a human activity and has all the follies of human activities. I think editors play a very important role in this. But they are also really overwhelmed with the number of manuscripts they handle, and they are handling manuscripts in very diverse areas. So, I think, it's almost an impossible task for them to ride herd over reviews that are unreasonable.

## NG

As an editor, I agree, it could be very challenging. I don't know if you know this, but in *Pathogens and Immunity*, we review articles submitted in any standard format, and we only require our particular publishing format after a submission is accepted for publication. Would you endorse that sort of approach for journals more widely?

## VD

Absolutely. It's common sense. And it would save tens of millions of dollars in wasted effort and much anguish. I, honestly, couldn't format a manuscript. I don't have the skills to do it. I see the people in the group struggling with it.

## NG

For those who wish to go into science, do you have any particular suggestions about what they need to know to go into a career in biomedical research?

## VD

I think a career in biomedical research can be very fulfilling, there is that thrill of discovery. There's really, for many people, no substitute. But it's imperative that one be absolutely passionate about it. And if one is not, it's not worth devoting one's time to a biomedical career. Because there are many bumps in the road. There's much adversity to overcome. And so, if you're passionate, and you enjoy the thrill of discovery, you will be more accepting of those bumps and adversity. But if you're not absolutely thrilled with discovery, you'll get frustrated. So I would say that people need to ask themselves – and there's no right answer or wrong answer; there's no good or bad – but, does it really drive them? And if it drives them, then great, but if it doesn't, then think of something else.

## NG

Finally, I've been devoted to medicine and science for many, many decades. Nevertheless, when I started hearing this term STEM and this constant drumbeat about focusing on science, technology, engineering, and math, all of which I think are great, but I don't see any reason to exclude other fields. So, I'm wondering how you look at that concept versus promoting a broader standard of liberal arts approach? Again, I don't think there's a one-size-fits-all formula, but in general, would you encourage people to focus on STEM subjects if they want a career in science or any of the related areas? Or would you counsel a broader range of academic and even non-academic experiences?

## VD

I'm convinced that broader is best. I favor a liberal arts education. I would do away with most AP classes. I'm quite sure that many students who take AP classes don't develop an in depth understanding of the complex material that's presented to them, but rather pass the test through a combination of rote learning and exercises. I may be a bit extreme on this, but I think a liberal arts education doing away with AP classes is the best way to go.

## NG

Well, that's a wonderful place to end. It's been a true thrill to have this conversation. I wish we could have had this conversation periodically for the last 40 years. I also have to say you're extremely articulate in expressing your views, and they reflect a great deal of thought, which is inspiring, quite frankly. Thank you again Vishva.

## Figures and Tables

**Figure:**